# Greater inhibition of female rat binge alcohol intake by adrenergic receptor blockers using a novel Two-Shot rat binge drinking model

**DOI:** 10.21203/rs.3.rs-4402198/v1

**Published:** 2024-05-29

**Authors:** Thatiane De Oliveira Sergio, Rebecca Jane Smith, Sarah E. Wean, Eric A. Engleman, Frederic W. Hopf

**Affiliations:** Indiana University School of Medicine, Department of Psychiatry, Indianapolis IN.

## Abstract

Binge drinking (BD) contributes strongly to the harms of alcohol use disorder. Most rodent models do not result in binge-level blood alcohol concentrations (BACs), and to better understand individual and sex differences in neurobiological mechanisms related to BD, the use of outbred rat strains would be valuable. Here, we developed a novel BD model where after 3+ months of intermittent access to 20% alcohol Wistar rats drank, twice a week, with two 5-minute intake (what we called Two-shot) separated by a 10-minute break. Our findings showed during Two-Shot that most animals reached ≥ 80mg% BAC levels (when briefly food-restricted). However, when increasing alcohol concentrations from 20% to 30%, 40%, or 50%, rats titrated to similar intake levels, suggesting rapid sensing of alcohol effects even when front-loading. Two-Shot drinking was reduced in both sexes by naltrexone (1mg/kg), validating intake suppression by a clinical therapeutic agent. Further, both propranolol (β adrenergic receptor antagonist) and prazosin (α1 adrenergic receptor antagonist) reduced female but not male BD at the lower dose. Thus, our results provide a novel model for BD in outbred rats and suggest that female binging is more sensitive to adrenergic modulation than males, perhaps providing a novel sex-related therapy.

## Introduction

Alcohol use disorder (AUD) is among the most prevalent mental diseases globally, and excessive alcohol drinking is now one of the leading causes of death, accounting for 1 in 5 deaths among 20–49 year old in the United States^1^. Binge drinking (BD), an episodic heavy use of alcohol, is a major obstacle to treating AUD^2–5^, and 3/4th of costs of binge intake are due to individuals who binge^2^. Reducing levels of BD can reduce health risks^3,6^ and incidence of relapse^4^, while BD can increase risk of developing more serious alcohol problems in non-dependent subjects^7–9^. In addition, rates of excessive alcohol drinking in women have risen dramatically in recent years^10–13^, and women have greater risk for alcohol problems including comorbidities^14–17^, and identification of sex-specific drivers would help personalize therapies to reduce drinking harms.

Thus, there is a considerable urgency to discover brain mechanisms that underlie BD, and this would be aided by rodent models with reliable high drinking levels. Several mouse lines show higher drinking and have provided many valuable insights^5^, while outbred rats would also have considerable utility when seeking to understand individual differences in alcohol drives. However, rats generally do not readily consume enough alcohol to reach high intoxication and blood alcohol concentrations (BAC) ≥ 80mg%, which meets the criteria of the National Institute on Alcohol Abuse and Alcoholism (NIAAA) for BD in humans^3,5,18^. During the past years, considerable advances were made using forced administration of alcohol through alcohol vapor, gavage, or liquid diet methods, and by studying strains with a genetic predisposition to alcohol (reviewed by Jeanblanc and colleagues^5^). In addition, hybrid operant models have provided useful BD paradigms in rats^5^ including sex differences^19,20^. Thus, we sought to develop a technically simple BD model using alcohol consumed from the bottle^21^, with the goal of setting a foundation to better understand individual and sex differences in underlying mechanisms, including where rats generally allow broader behavioral investigations.

Another useful feature of a BD model would be to detect changes after a long-term drinking history, as often occurs in humans. We and others have used an intermittent access two-bottle choice paradigm (IA2BC), where, after 3 + months of alcohol intake, animals exhibit several features related to human AUD including escalation^22,23^, sensitivity to drugs that reduce human drinking^22,24,25^, withdrawal signs (although moderate)^26,27^, front-loading^5,21,28–30^, and compulsive intake (consumption despite negative consequences^31,32^). However, in this model, BAC levels range around 40–60mg% in 20–30 minutes^22,33^, suggesting need for a paradigm where binge-level intake is achieved.

Male and female Wistar rats have a rapid initial intake after IA2BC (front loading) with around 80% of alcohol consumption occurring during the first 5 minutes of exposure^28–30^, and others have noted the importance of rate of intake for rodent BD models^5^. Based on this evidence, we sought to develop a novel BD animal model where we restricted alcohol access and determined whether giving rats two 5-minute alcohol access periods, separated by 10 minutes (which we call the Two-Shot model), along with increasing the standard 20% alcohol to 30%, 40%, or 50%, would lead to higher BACs and thus binge intake levels. Importantly, most animals reached ≥ 80mg% BAC levels during Two-Shot, providing a novel behavioral model to assess mechanisms of BD. In addition, and surprisingly, rats titrated to similar alcohol intake levels despite the increases in alcohol concentration (across 20–50%), suggesting rapid sensing of alcohol effects. Also, to pharmacologically validate the Two-Shot model, we found that alcohol intake was significantly reduced in both sexes by naltrexone, a drug used to treat AUD^34^. Finally, due to the urgent need to develop novel pharmacotherapies for problem drinking, we investigated the impact of α1 adrenergic receptor (AdrR) antagonists and β AdrR antagonists through injections of prazosin and propranolol, respectively. Inhibiting α1 AdrRs reduces several aspects of drive for alcohol in humans, and represent a critical new therapeutic against excessive consumption^25,35^ (detailed in Discussion). β AdrR inhibitors have also shown efficacy against aspects of alcohol behavior in humans but have been understudied (see Discussion). Interestingly, a lower dose of propranolol or prazosin decreased BD only in females, with no effects in males, while higher dose of prazosin or propranolol decreased BD in both sexes. Taken together, our results showed that giving rats 3 + months of intermittent intake, then giving rats restricted access with two brief exposures of alcohol each drinking day, can be used as a new model of rat binge drinking.

## Results

### The Two-Shot drinking model

**Figure 1:**
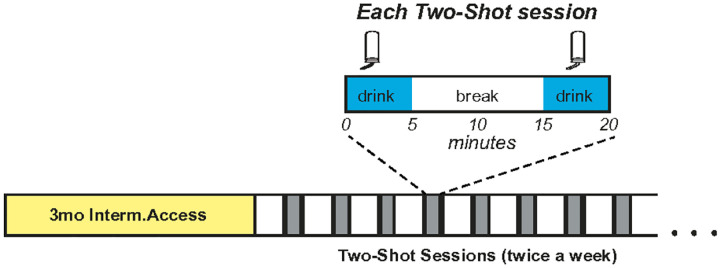
Schematic representation of the novel Two-Shot model. After 3+ months of IA2BC, rats started to drink twice a week in the Two-Shot model. In each session of the Two-Shot, rats drank for 5min (Shot-1), followed by 10 min of break, and then a second 5-min drinking (Shot-2).

We previously found both sexes of Wistar rats have strong initial intake in a drinking session, also called front-loading^28–30^. To increase intake in outbred rats to BD levels, we developed a new restricted-access paradigm. Male and female Wistar rats first drank for ~ 3 months under IA2BC, and then switched to drinking twice a week in a Two-Shot model. In each Two-Shot drinking session (Fig. 1), rats had 5 minutes of access to alcohol (called “Shot-1”), followed by a 10-minute break, and then had an additional 5 minutes access to alcohol (called “Shot-2”). Thus, each Two-Shot session was a total of 20 minutes.

### Two-Shot alcohol led to binge-level BACs

We first evaluated whether consumption of 20% alcohol during the Two-Shot session would lead to binge-level BACs (≥ 80mg%). Thus, male (n = 14) and female (n = 18) Wistar rats drank 20% alcohol in a Two-Shot session, and blood was collected through a saphenous vein 20 minutes after ending the Shot-2. For these studies, rats were food restricted for 3 hours before the Two-Shot session, to remove variability associated with different amounts of food in the gut. While BAC without food restriction would be more variable, it is likely that rats would experience these higher level BACs on some intake days, especially since rats with ad-libitum access to food showed strong titration of intake levels when alcohol concentration was altered (following section).

**Figure 2:**
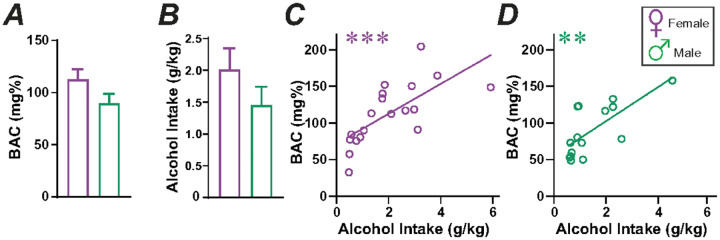
Two-Shot model with 20% alcohol leads to binge-level BACs. **(A)** BAC was greater than 80mg% with 20% alcohol drinking in females (purple) and males (green) and **(B)** alcohol intake >1g/kg. Also, alcohol intake level (g/kg) and BACs were significantly correlated in females **(C)** and males **(D)**. * p<0.05; **p<0.01; ***p<0.001.

Thus, these studies give an estimate of the maximum possible BAC that could be reached. Our findings showed that BAC was greater than 80mg% for both sexes (Fig. 2A, female: 112.9 ± 9.6 mg%, male: 89.8 ± 9.2 mg%), with intake > 1g/kg in 20 minutes (Fig. 2B). In addition, alcohol intake level (g/kg) and BACs were significantly correlated in both females (Fig. 2C; F_(1,17)_ = 16.16, *p* = 0.0009, R^2^ = 0.4874) and males (Fig. 2D; F(1,13) = 13.41, *p* = 0.0029, R^2^ = 0.5078), with no sex difference in slopes (F_(1,30)_ = 0.114, *p* = 0.7381). Male intake-BAC relations remained significant without the largest outlier value (n = 13, F_(1,12)_ = 5.687, *p* = 0.0345, R^2^ = 0.3215). Thus, during Two-Shot model of 20% ethanol, both sexes exhibited high drinking levels and reached BACs greater than 80mg%.

### Rats maintained similar intake levels as alcohol concentration increased

Since rats show rapid initial intake (front-loading) during our typical 20min/day, 5d/week intake paradigm (after the 3 + months of IA2BC)^28–30^, we considered the possibility that increasing the alcohol concentration from standard 20–30%, 40%, or 50% alcohol would lead to even higher BAC levels (assuming the same volume of intake), increasing the chance of exceeding binge-level BACs. Indeed, front-loading rats seem to consume alcohol faster than significant levels of alcohol can enter the bloodstream^36,37^. Thus, we examined n = 20 animals per sex, with each rat being exposed to each alcohol concentration at least twice, in randomized order across drinking days. Surprisingly, we found that drinking levels were similar regardless of alcohol concentration, although female consumption was higher than males. This perhaps suggests that rats have early interoceptive changes which they use to titrate to similar intoxication levels, regardless of the concentration of alcohol consumed (and see Discussion^38^).

**Figure 3:**
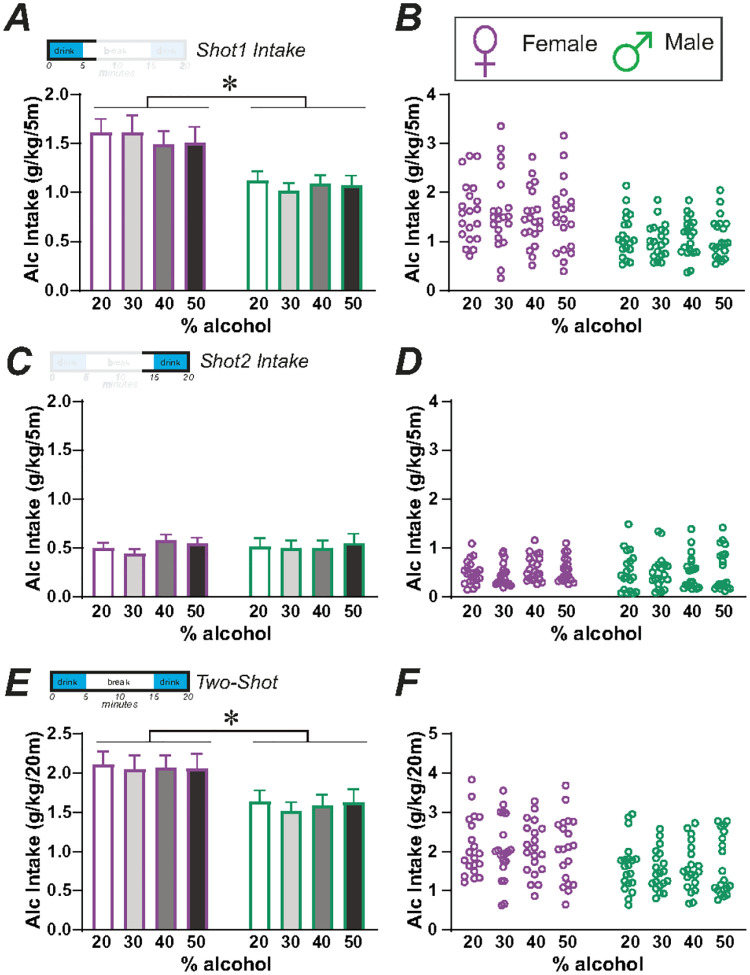
Female and male rats maintained similar intake levels as alcohol concentration increased. **(A,B)** During Shot-1, females consumed more alcohol than males and, for both sexes, there was no difference in intake levels between 20%, 30%, 40%, or 50% alcohol. (**C,D**). For Shot-2, there were no differences by sex or alcohol concentrations. (**E,F**) During the full Two-Shots intake period, females consumed more alcohol, and there was no difference between the alcohol concentrations. n=20 per sex *p<0.05

Thus, a two-way repeated-measures ANOVA for Shot-1 (Fig. 3A,B) showed a main effect of sex (F_(1,38)_ = 9.868, *p* = 0.0033), but no effect for alcohol concentration (F_(2.224,84.50)_ = 0.467, *p* = 0.6489) or interaction (F_(3,114)_ = 0.632, *p* = 0.5958). Thus, within the first 5-min intake period, rats had similar intake levels (g/kg) even when consuming different alcohol concentrations. However, we note that Shot-2 consumption (Fig. 3C,D) showed no effect of sex (F_(1,38)_ = 0.000, *p* = 0.9894), alcohol concentration (F_(2.909,110.5)_ = 0.594, *p* = 0.6153), or interaction (F_(3,114)_ = 0.373, *p* = 0.7724). Also, Shot-1 intake was 2–3x greater than Shot-2 drinking, consistent with front-loading. These suggest that titration of intake volume across different alcohol concentrations occurred within Shot-1, the first 5-min access period. Further, the analysis for Two-Shot session intake (Shot-1 plus Shot-2) (Fig. 3E,F) showed a main effect of sex (F_(1,38)_ = 7.013, *p* = 0.0117) but not alcohol concentration (F_(2.509,95.35)_ = 0.253, *p* = 0.8247) or interaction (F_(3,114)_ = 0.077, *p* = 0.9723). Thus, rats overall maintained a similar average drinking level across different alcohol concentrations during the Shot-1 period. While these results suggested that increasing alcohol concentrations was not effective in further increasing drinking levels, they also indicated that rats may have some type of rapid interoception, which they utilize to titrate their drinking levels to approximately some preferred level in each session.

**Figure 4:**
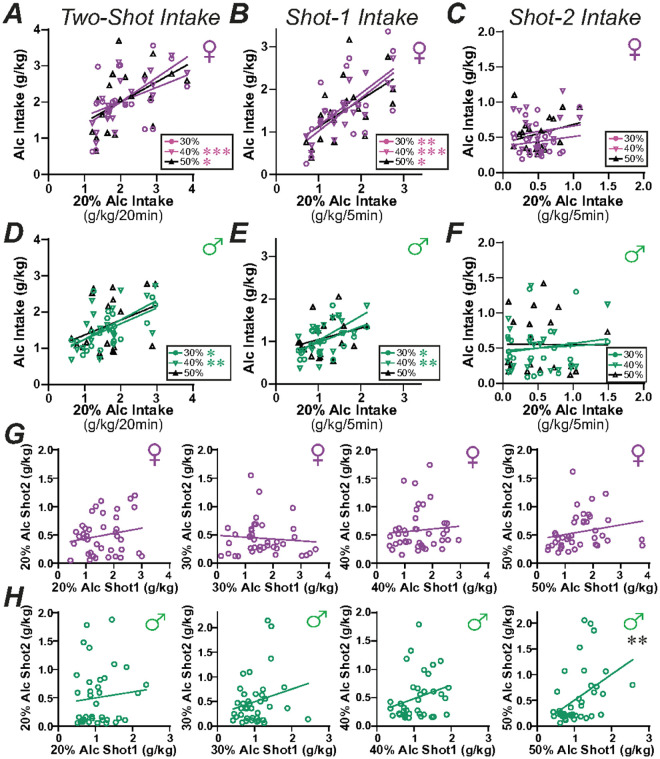
Relation of 20% alcohol intake to intake of other alcohol concentrations. **(A-C)** Relation of female intake level for 20% alcohol to higher alcohol concentrations, for **(A)**Two-Shot, **(B)** Shot-1 alone, and **(C)** Shot-2 alone. **(D-F)** Relation of male intake level for 20% alcohol to higher alcohol concentrations, for **(D)**Two-Shot, **(E)** Shot-1 alone, and **(F)** Shot-2 alone. **(G,H)** Relation between Shot-1 and Shot-2 intake, for each alcohol concentration separately, in (G) females and (H) males. * p<0.05; **p<0.01; ***p<0.001.

To further evaluate if rats were titrating intake to reach a particular level, we examined whether intake level with 20% alcohol would correlate with consumption with higher alcohol concentrations. In females, 20% alcohol consumption levels correlated with 40% and 50%, although not 30%, during the full Two-Shot period (Shot-1 plus Shot-2) (Fig. 4A; **30**%: F_(1,18)_ = 2.912, *p* = 0.1051, R^2^ = 0.1393; 40%: F_(1,18)_ = 21.94, *p* = 0.0002, R^2^ = 0.5493; 50%: F_(1,18)_ = 6.039, *p* = 0.0244, R^2^ = 0.2512). Also, in females, 20% intake in Shot-1 correlated with Shot-1 drinking for all other alcohol concentrations (Fig. 4B; **30**%: F_(1,18)_ = 9.937, *p* = 0.0055, R^2^ = 0.3557; 40%: F_(1,18)_ = 39.79, *p* < 0.0001, R^2^ = 0.6885; 50%: F_(1,18)_ = 8.003, *p* = 0.0111, R^2^ = 0.3078). In males, intake level with 20% alcohol correlated with drinking levels with 30% and 40% for the full Two-Shot period (Fig. 4D; **30**%: F_(1,18)_ = 7.910, *p* = 0.0115, R^2^ = 0.3053; 40%: F_(1,18)_ = 8.897, *p* = 0.0080, R^2^ = 0.3308), and for Shot-1 alone (Fig. 4E; **30**%: F_(1,18)_ = 5.130, *p* = 0.0361, R^2^ = 0.2218; 40%: F_(1,18)_ = 12.22, *p* = 0.0026, R^2^ = 0.4043). In males, 20% intake did not correlate with 50% consumption levels (Two-Shot: F_(1,18)_ = 2.552, *p* = 0.1276, R^2^ = 0.1242, although significant without one outlier F_(1,17)_ = 5.477, *p* = 0.0317, R^2^ = 0.2437; Shot1: F_(1,18)_ = 1.547, *p* = 0.2295, R^2^ = 0.0792). In strong contrast, Shot-2 20% intake did not correlate with Shot-2 drinking of any other alcohol concentrations in females (Fig. 4C; **30**%: F_(1,18)_ = 0.096, *p* = 0.7607, R^2^ = 0.0956; 40%: F_(1,18)_ = 0.418, *p* = 0.5263, R^2^ = 0.0227; 50%: F_(1,18)_ = 0.002, *p* = 0.9666, R^2^ = 0.0001) or males (Fig. 4F; **30**%: F_(1,18)_ = 0.399, *p* = 0.5356, R^2^ = 0.0217; 40%: F_(1,18)_ = 0.420, *p* = 0.5251, R^2^ = 0.0228; 50%: F_(1,18)_ = 1.315, *p* = 0.2665, R^2^ = 0.0681). Thus, these results suggest that, even with briefer intake periods, rats titrated the amount of alcohol consumed, regardless of alcohol concentration.

Since rats consumed similar g/kg levels across alcohol concentrations, whether across the 20-minute Two-Shot period or during Shot-1, and females consumed more alcohol for Shot-1 but not Shot-2 (Fig. 3), titration of intake seemed to primarily occur with Shot-1. In this case, consumption in Shot-2 would notcorrelate with drinking in Shot-1. Alternately, rats that drank less in Shot-1 might increase intake in Shot-2 to bring intake nearer to an optimal level. However, Shot-2 intake level did not correlate with Shot-1 level for any alcohol concentration in females (Fig. 4G; **20**%: F_(1,37)_ = 1.353, *p* = 0.2522, R^2^ = 0.0353; 30%: F_(1,37)_ = 0.292, *p* = 0.5923, R^2^ = 0.0078; 40%: F_(1,38)_ = 0.290, *p* = 0.5934, R^2^ = 0.0076; 50%: F_(1,38)_ = 1.885, *p* = 0.1778, R^2^ = 0.0473). In males (Fig. 4H), there was no Shot-1 versus Shot-2 correlation for 20% (F_(1,35)_ = 0.314, *p* = 0.5786, R^2^ = 0.0089), 30% (F_(1,37)_ = 1.925, *p* = 0.1736, R^2^ = 0.0495), or 40% (F_(1,35)_ = 2.524, *p* = 0.1211, R^2^ = 0.0673), although 50% Shot-2 intake was positively correlated with Shot-1 (F_(1,37)_ = 8.522, *p* = 0.0059, R^2^ = 0.1872). Thus, alcohol titration predominantly occurred within the first 5-minute access period.

### Female drinking was inhibited by lower concentrations of adrenergic receptor blockers relative to males

Next, to pharmacologically validate the Two-Shot model, we first tested naltrexone, a drug often used to treat AUD^22,34^. Thus, we administered 1mg/kg naltrexone, a dose that reduces alcohol intake in rodents^22^. In addition, we examined the effect of prazosin, antagonist of a1 adrenergic receptor, and propranolol, the β adrenergic receptor antagonist, as we^24,39^ and others^40,41^ have examined in previous preclinical studies.

**Figure 5:**
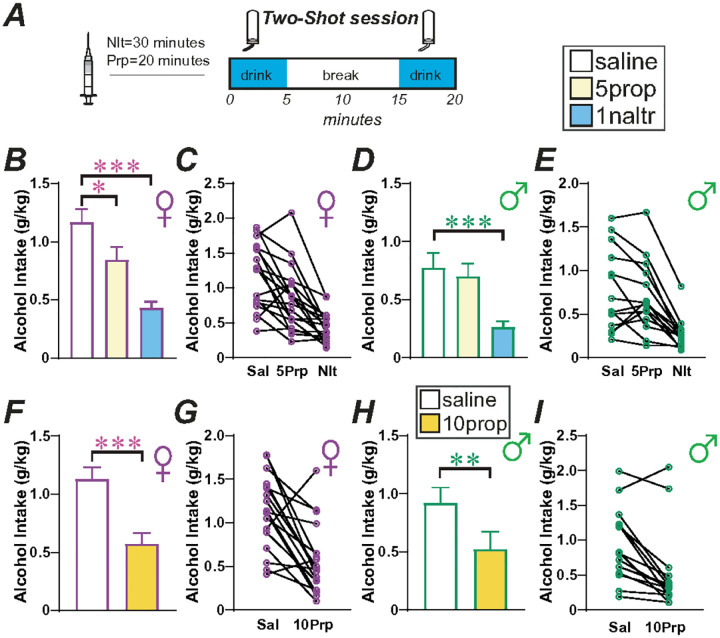
Two-Shot intake was inhibited by naltrexone in both sexes, and females drinking was inhibited by lower concentrations of propranolol. **(A)** Cartoon of injection timing. **(B-E)** Impact of 1mg/kg naltrexone or 5mg/kg propranolol in **(B,C)** female and **(D,E)** male rats. **(F-I)** Impact of 10mg/kg propranolol in **(F,G)** female and **(H,I)** male rats. * p<0.05; **p<0.01; ***p<0.001, vs group control.

First, we compared the impact of 5mg/kg propranolol and 1mg/kg naltrexone versus saline vehicle in 18 females and 15 males using a within-rat, randomized design. In females, there was a significant effect by one-way repeated-measures ANOVA (Fig. 5B, C; F_(1.932,32.84)_ = 28.23, *p* < 0.0001), with post-hoc differences between saline and naltrexone (*p* < 0.0001) and between saline and 5mg/kg propranolol (*p* = 0.0206). In males, there was a signi cant effect by one-way repeated-measures ANOVA (Fig. 5D,E; F_(1.702,22.12)_ = 22.21, *p* < 0.0001), with post-hoc differences between saline and naltrexone (*p* = 0.0005) but not between saline and 5mg/kg propranolol (*p* = 0.5471). Thus, naltrexone reduced Two-Shot intake in both sexes, providing pharmacological validation of the utility of this novel BD model. However, 5mg/kg propranolol only inhibited drinking in the Two-Shot model in female rats. In a second set of experiments in the same rats, we increased the dose of propranolol to 10mg/kg to verify if we could also decrease drinking in males^39,40^, compared to vehicle. This higher dose significantly reduced BD in females (Fig. 5F,G; t_(17)_ = 4.546, *p* = 0.0003) and males (Fig. 5H,I; t_(14)_ = 4.107, *p* = 0.0011) (both paired t-test saline vs 10mg/kg within-rat). Thus, the higher tested dose of propranolol reduced alcohol drinking in both sexes, but the lower propranolol dose tested only impacted female binge intake.

**Figure 6:**
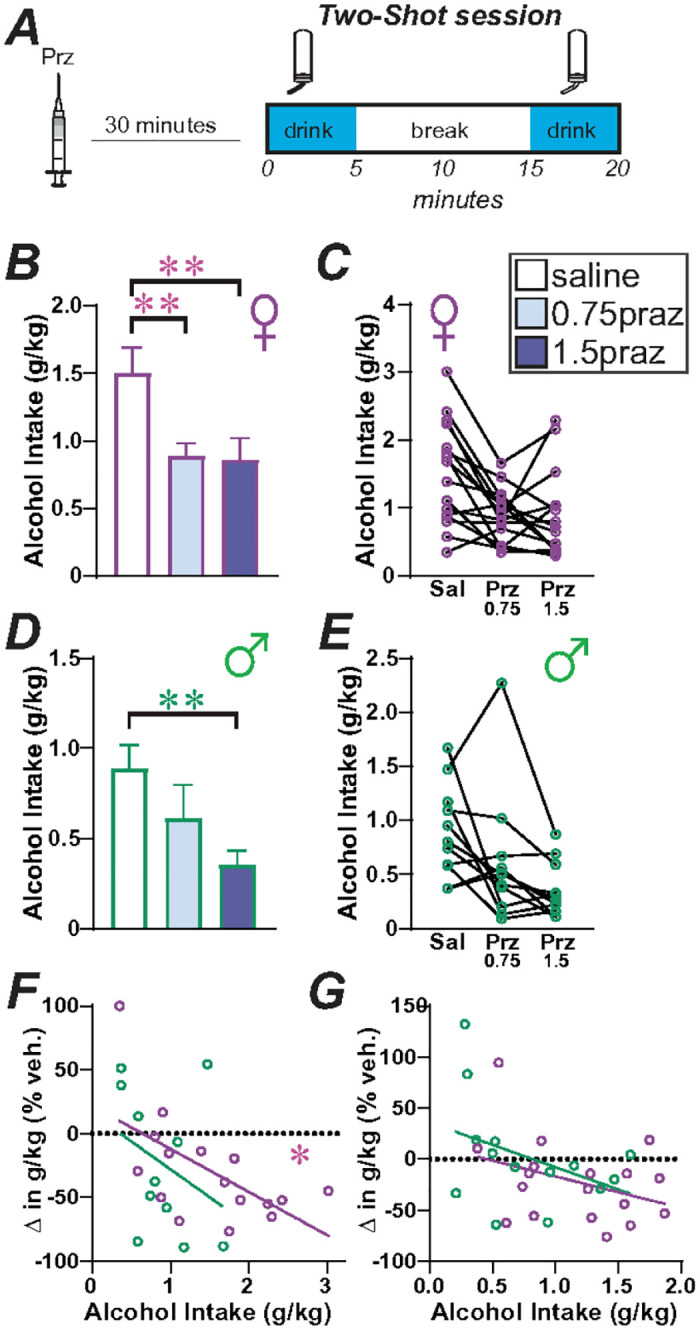
Female drinking was inhibited by lower concentrations of prazosin relative to males. **(A)** Cartoon of injection timing. **(B-E)** Impact of 0.75mg/kg or 1.5mg/kg prazosin on **(B,C)** female and **(D,E)** male rat Two-Shot drinking. **(F,G)** Relationship between basal intake levels and impact of **(F)** 0.75mg/kg prazosin or**(G)** 5mg/kg propranolol on drinking (change in drinking determined as drug/vehicle intake and expressed as percent). * p<0.05; **p<0.01.

We next tested if the α1 adrenergic inverse agonist, prazosin could impact 20% alcohol consumption in the Two-Shot model. For this, we compared 0.75 mg/kg and 1.5mg/kg prazosin versus saline vehicle in 16 females and 12 males, in randomized order within and across rats. In females, there was a significant effect by one-way repeated-measures ANOVA (Fig. 6B,C; F_(1.972,29.57)_ = 11.13, *p* = 0.0003), with post-hoc significant effects of both 1.5mg/kg (*p* = 0.0036) and 0.75 mg/kg (*p* = 0.0024) prazosin. In males, there was a significant effect by one-way repeated-measures ANOVA (Fig. 6D,E; F_(1.460,14.60)_ = 6.581, *p* = 0.0141), with post-hoc significant effects of 1.5mg/kg prazosin (*p* = 0.0026) but not for 0.75 mg/kg prazosin (*p* = 0.3326). Thus, the higher dose of prazosin tested reduced drinking in both sexes, while the lower dose of prazosin tested only decreased binge alcohol consumption in females.

Taken together, prazosin and propranolol were effective at reducing excessive alcohol consumption in females at lower doses than those that were effective in males. However, since females drank more alcohol than males, another possibility is that these lower tested doses of prazosin and propranolol reduced intake only in higher drinkers. For 0.75mg/kg prazosin, higher drinkers did show a greater prazosin reduction in drinking, which was significant in females (Fig. 6;F_(1,14)_; F = 7.592, *p* = 0.0155, R^2^ = 0.352) with a similar trend but non-significant in males (Fig. 6F; F(1,9) = 1.140, *p* = 0.3134, R^2^ = 0.113). For 5mg/kg propranolol, basal intake did not relate to propranolol reduction in drinking in females (Fig. 6G; F_(1,16)_ = 2.323, *p*= 0.1470, R^2^ = 0.127), males (Fig. 6G; F_(1,14)_ = 2.175, *p* = 0.1660, R^2^ = 0.154). Thus, propranolol might re ect a more sex-selected target to reduce female drinking, while prazosin affected higher drinkers.

## Discussion

Since most rat alcohol models with voluntary intake do not result in binge-level intake, we sought to develop a novel, restricted-access paradigm. Thus. after 3 + months of IA2BC, rats switched to drinking twice per week in a Two-Shot model, with two 5-minute intake periods, separated by 10 minutes break, in each drinking session. To test BACs, rats were briefly food-restricted to reduce the impact of food in gut and to give the maximum possible BAC. Most animals reached ≥ 80mg% BAC levels during Two-Shot, providing a novel model to assess BD mechanisms. Since our rats rapidly front-load, we also reasoned that increasing alcohol concentrations (from 20–30%, 40%, or 50%) might lead to even higher intake levels. Surprisingly, rats titrated intake of different alcohol concentrations to similar g/kg drinking levels in the first 5-minute intake period (Shot-1), suggesting that they rapidly sensed some aspect of alcohol effects even when front-loading. Nonetheless, to pharmacologically validate the Two-Shot model, we found drinking was reduced in both sexes by naltrexone, a human therapeutic agent for problem drinking. In addition, blocking β adrenergic receptors (with propranolol) or α1 adrenergic receptors (with prazosin) reduced drinking in a sex-specific manner according with the dose used. Thus, our results provide a novel model for BD in outbred rats, and suggest that female binge intake is more sensitive to adrenergic modulation than males.

We note that we did not test BACs without food deprivation, and thus BACs under ab libitum conditions would be somewhat lower. However, it is likely that rats by chance would drink with minimal food in gut on some days, and thus could come to learn the association between intake and intoxication. In addition, we surprisingly found that varying the concentration of alcohol consumed, across 20–50%, did not alter the g/kg level of intake. This was unexpected, since our rats have strong, rapid initial intake (front-loading)^28–30^, and dialysis studies have found that, after front loading, alcohol takes several minutes to enter the blood stream in significant levels^36,37^. Thus, we predicted a priori that rats would consume similar volumes of alcohol, before strong bodily and intoxication effects would occur. Rats might also change intake levels based on alcohol taste, although our other studies find that rats exhibit aversion-resistant intake (continued consumption even with the bitter quinine in the alcohol)^24,28–30,33,39^. Instead, we found that drinking levels were similar across the different alcohol concentrations tested (20%, 30%, 40%, 50%), although females had higher intake than males for each concentration. This suggests that rats have some, as yet unidentified, mechanism for sensing their alcohol intake level. Considerable future studies will be needed to understand these findings. We also note that rats self-administering different higher concentrations of alcohol under operant methods also titrate to similar drinking levels^38^. While such operant methods have slower rates of intake, relative to bottle-based drinking, our findings agree with previous studies suggesting that responding for alcohol reflects “motivation to obtain a specific pharmacological effect of ethanol”^38^.

Previous work has put considerable effort into developing rat models which lead to binge-level intake. Certainly C57BL/6 mice are widely observed to reach binge-level BACs^42^, and thus are a valuable model, but rats have generally been considered to afford easier training in a wider range of behavioral paradigms. Alcohol dependence, through use of repeated alcohol vapor exposure or liquid diet, can lead to high alcohol intake in rats (reviewed in^5^). Other studies have utilized rat lines genetically selected for high alcohol preference or intake^43^. The intermittent access model we and others use (IA2BC) can lead to withdrawal signs in rats, although moderate^26,27^. However, some studies have highlighted how many problem drinkers do not exhibit dependence^7^. Our goal was to develop a BD model in outbred rats to facilitate the identification of individual and sex differences in drive for alcohol. It is important to note that others have developed useful binge operant intake models in rats^5^, which can have sex differences in their impact^19,20^. While there is some consideration of which rat strain might be optimal for BD studies^44^, as discussed by Sauton and colleagues^44^, rats of a given strain can differ among vendors, complicating identification of an widely usable optimal model. Indeed, having several BD models in outbred rat lines will likely accelerate and better validate our understanding of BD mechanisms.

We also examined the pharmacological sensitivity of female and male binge-level alcohol intake. There is a critical need for better therapies, especially drugs that are already FDA approved and could be quickly repurposed^34,45^. Prazosin and related compounds have been of particular interest since prazosin reduces drinking and craving in humans with drinking problems^25^. The involvement of β AdRs for human problem drinking has received limited study in recent times^45,46^, although early clinical studies found that β AR antagonists can reduce symptoms related to alcohol withdrawal and cravings^47–50^. Here, we found that lower tested doses of prazosin and propranolol reduced BD in females but not males. Prazosin decreases intake in dependent rats at lower doses (0.5–1.0mg/kg) than are effective in non-dependent rats (1.5–2.0mg/kg)^41^, while 5 and 10mg/kg propranolol decreases operant responding for alcohol in dependent male rats, with only 10mg/kg affecting non-dependent rats^40^, and dependence produces changes in β AdR function that promote intake^51^. Thus, our findings support the possibility that female drinking may be preferentially impacted by AdR modulation. Indeed, prazosin is more effective in human drinkers with greater withdrawal anxiety^25^, and women more often report drinking alcohol to cope with stress and anxiety^17,52^.

This study has some limitations. First, we did not examine estrous cycle: once an addiction-related state is established, alcohol intake level is often unrelated to estrous stage^53–55^, including in our studies^30^. In addition, we did not test the impact of prazosin and propranolol on sweet fluid intake, to control for potential non-specific effects. However, prazosin does not impact saccharin intake or locomotion in our rats^24^, similar to the lack of prazosin effects on sugar^56^ or water^41,57^ intake, or locomotion^41,57,58^, and the lack of effect of propranolol on saccharin^59^ or sugar^60^ intake.

In summary, our findings showed that alcohol intake in Wistar rats under the Two-Shot paradigm can lead to BAC levels ≥ 80%, which is reduced by the human AUD drug naltrexone. Moreover, propranolol and prazosin reduced drinking in females at lower doses than in males, suggesting that female BD is more sensitive to adrenergic modulation than males.

## Materials and Methods

### Animals and alcohol drinking methods

All experimental procedures were conducted in strict accordance with the Guide for Care and Use of Laboratory Animals provided by the National Institutes of Health and approved by the Institutional Animal Care and Use Committee of Indiana University. Additionally, all methods and results were described following Animal Research: Reporting In Vivo Experiments Arrive. A total of 30 female and 33 male Wistar rats arrived at 45–50 days old and were singly housed, with both sexes in the same housing room in a reverse dark-light cycle (lights off 11 am-11 pm). All behavioral tests happened in a dark cycle. After 2wk acclimation, rats began access to alcohol (20% v/v diluted in water) in the intermittent two-bottle choice paradigm (IA2BC), which involves intake of 20% alcohol vs water-only for 18–24 hours starting on Monday, Wednesday, and Friday at ~ 1hr into the dark cycle^22^, as we used before^24,28–30,33,39^. After 3 + months IA2BC, we switched rats to drink twice per week in the Two-Shot model (Fig. 1): in each Two-Shot session, rats had access to drink for 50min (Shot-1), followed by 10 min of break and, then a second 5-min drinking (Shot-2). Rats were allowed 2–3 months of Two-Shot drinking before experiments began.

For comparison of drinking levels across 20%, 30%, 40%, and 50% alcohol concentrations (Figs. 3,4), 20 rats of each sex were tested; for most rats, two drinking sessions for each alcohol concentration were assessed in randomized order across rats and alcohol concentrations. Data were then averaged for each alcohol concentration for each rat, as we have done before to reduce variability in drinking values^24,33,39^. These studies were in a different cohort of rats from those tested with pharmacological agents.

### Blood Alcohol Concentration

After testing intake with different alcohol concentrations, these same rats were used to determine BACs. One week before starting blood collection, rats were gently handled by the experimenter, once a day, for ~ 5 min. This was performed after alcohol concentration studies, to reduce potential stress effects on drinking studies. BAC was collected from the saphenous vein 20 minutes after the second Shot of alcohol at 20% and 50% concentrations. Rats were food-restricted for 3 hours before drinking, to allow determination of maximum possible BACs. For all other studies, rats were not food restricted, and thus had ad-libitum access to food. BAC concentrations were determined, relative to alcohol standards, by gas chromatography as described in^22,36^.

### Drugs

Propranolol hydrochloride was from Tocris. Prazosin hydrochloride and naltrexone hydrochloride were from Sigma-Aldrich (USA). All drugs were dissolved in sterile saline (0.9%), except prazosin which was dissolved in sterile water (as we did before^24^). All drugs were prepared the same day they were used for experiments.

### Effect of pharmacological agents on Two-Shot drinking

Rats were exposed to each pharmacological treatment in a within-subject design. Groups were randomized across animals before the beginning of the test sessions, and all conditions were balanced to make sure that all groups were tested on the same test day. Before test sessions, rats were handled by the experimenter, with 1–3 sessions of habituation to vehicle injection, to avoid stress reactions during the injection procedure. Since propranolol and naltrexone had the same vehicle (saline), we ran both treatments in the same cohort to reduce the number of animals. Thus, propranolol 5mg/kg (n = 18 females; Fig. 5B, and 15 males; Fig. 5D) was injected 20 minutes before the beginning of the Two-Shot session, while naltrexone 1mg/kg (Fig. 5D) or vehicle was injected 30 minutes before drinking (Fig. 5A). Afterwards, the same rat cohort was tested with propranolol 10mg/kg vs vehicle (Fig. 5F,H) 20 minutes before Two-Shot session Times for administration and doses of propranolol was chosen by our previous work^39^ and naltrexone dose was from Simms and colleagues^22^.

After propranolol/naltrexone testing, these same rats were tested with prazosin. These rats were given ~ 1 month of Two-Shot drinking in case of disruptions in drinking due to i.p. injections, and in this time two male and two female rats died. Prazosin was tested at the doses of 0.75mg/kg or 1.5mg/kg, or vehicle (Fig. 6B–F), and injected 30 minutes before the beginning of the Two-Shot session (Fig. 6A). The doses of prazosin were chosen by our previous work^24^.

### Data Analysis

Alcohol consumption was determined through changes in bottle weight before and after a drinking session and converted to grams alcohol/kilograms body weight. Statistical comparisons were primarily performed in a within-subject design. Data were mostly analyzed by one- or two-way ANOVA with repeated measures followed by the Bonferroni test, while some comparisons used paired t-tests. Statistical analysis was performed using GraphPad Prism. All data are shown as mean ± SEM.
